# Comparative Transcriptome Analysis Reveals the Regulation of Growth Activity in *Poria cocos* Under Different Light Duration Stimulations

**DOI:** 10.3390/genes16121404

**Published:** 2025-11-24

**Authors:** Chengwen Wu, Shanwen Ye, Xuhui Wei, Rong Zheng

**Affiliations:** 1 Youxi Forestry Science and Technology Promotion Center, Sanming 365100, China; lygwcw@126.com; 2 Fujian Academy of Forestry, Fuzhou 350012, China; yeshanwen_faf@163.com; 3 Jinshan Campus, Fujian Agriculture and Forestry University, Fuzhou 350002, China; 17847699436@163.com

**Keywords:** *Poria cocos*, light exposure time, *BLI* gene, growth activity regulation, transcriptome sequencing

## Abstract

**Background**: Light is an important environmental signal that regulates the growth and metabolism of fungi. This study aims to reveal the molecular regulatory mechanism of different light durations on the growth activity of *Poria cocos*. **Methods**: By setting up three groups of light treatment: 0 days (sample 1), 15 days (sample 2), and 30 days (sample 3), and combining transcriptome sequencing (RNA-seq) with qRT-PCR for verification, the effects of light on the gene expression of *Poria cocos* (*Poria cocos (Schw.) Wolf*) were systematically analyzed. **Results**: A total of 4332 differentially expressed genes (DEGs) were identified in this study. Among them, the blue light-responsive genes, *BLI-3* and *BLI-4*, were significantly upregulated at the DT15 stage, reaching 576.08 times and 31.30 times, respectively, while they were sharply downregulated at the DT30 stage. The KEGG enrichment analysis revealed that the DEGs were mainly involved in secondary metabolite synthesis, carbon metabolism, amino acid synthesis, redox reactions, and the MAPK signaling pathway. At the DT15 stage, genes related to growth metabolism, such as *CYP*, *SNF1*, and *COX*, were highly expressed, indicating active metabolism at this stage. However, in the DT0 and DT30 stages, ROS-related genes such as NADPH-dependent oxidoreductases were upregulated, leading to oxidative stress damage and inhibiting growth. Additionally, the high expression of *BLI-3* and *BLI-4* significantly activated ergosterol synthesis genes, enhancing cell membrane stability. The WGCNA co-expression network analysis revealed a high degree of correlation between *BLI-4* and *MAPKKK* and *CYP* genes and proposed a potential “*BLI-4-MAPKKK-CYP*” regulatory axis, providing insights into the molecular pathway by which light regulates the metabolism and homeostasis of *Poria cocos*. **Conclusions**: This study has for the first time systematically revealed the molecular mechanism by which light duration regulates the growth activity of *Poria cocos*. It has clarified the core role of the *BLI* gene family in light signal perception and metabolic regulation. It has also elucidated the molecular pathways by which light regulates the synthesis of ergosterol, energy metabolism, and oxidative stress response in *Poria cocos*. This provides innovative theoretical support for optimizing the light regulation strategies in *Poria cocos* cultivation and also offers important references for the study of environmental response mechanisms in other medicinal fungi.

## 1. Introduction

Fungi play a crucial role in both ecological systems and the pharmaceutical field, with *Poria cocos* (*Poria cocos (Schw.) Wolf*) receiving considerable attention as an important medicinal species [1]. *Poria cocos*, an edible and medicinal fungus, is derived from the sclerotium of the Polyporaceae family. This species is predominantly found in the humid subtropical regions of East Asia, including China, Vietnam, and the Philippines [2,3]. It develops symbiotically on the roots of Pinus species, forming sclerotia—compact masses of hardened fungal mycelium that function as storage structures for nutrients. These sclerotia are widely used in medicine due to their diverse pharmacological properties [4]. *Poria cocos* possesses diuretic, spleen-strengthening, and sedative effects, and has also been shown to exhibit notable anti-inflammatory, antitumor, antioxidant, and immunomodulatory activities [5,6,7,8,9].

In higher plants, light serves not only as the energy source for photosynthesis but also as a pivotal environmental signal that regulates gene expression networks through sophisticated photoreceptor systems. *Arabidopsis thaliana* and other higher plants perceive light signals via multiple classes of photoreceptors, including phytochromes [10] and cryptochromes [11]. These receptors directly activate the expression of over 2000 light-responsive genes by modulating the stability of transcription factors such as *HY5*. This multi-layered regulatory mechanism enables plants to precisely coordinate photosynthetic metabolism, morphogenesis, and seasonal adaptive development [12,13]. In contrast to the dual-pathway energy-signal utilization strategy of plants, fungi, as heterotrophic organisms, have evolved a unique light information perception system. Filamentous fungi, represented by *Trichoderma*, perceive photoperiodic changes through blue light receptors such as the White Collar complex [14]. These light signaling systems do not participate in energy capture but optimize environmental adaptability by regulating secondary metabolite synthesis and carbon-nitrogen substrate utilization preferences. Notably, photoperiod length significantly influences fungal conidiation rhythms. In *Neurospora crassa*, the periodic splicing of the circadian clock gene frequency (frq) is directly regulated by light–dark cycles, driving the circadian oscillation of approximately 40% of metabolic enzymes, indicating deep integration of light signaling into fungal development and metabolic networks [15,16,17]. For fungi, the duration of light exposure plays a crucial role in multiple life processes, including growth, reproduction, and gene expression [18,19].

Light serves as a critical environmental signal that significantly influences fungal growth, development, and environmental adaptability by regulating physiological metabolism, energy status, and light-responsive gene expression [20]. Research demonstrates that varying light qualities can substantially alter fungal biomass and secondary metabolite synthesis. Under complete darkness, both biomass and intracellular/extracellular pigment production in five pigment-producing filamentous fungi were markedly enhanced. Notably, *Neurospora crassa* exhibited a red pigment yield of 36.75 ± 2.1 OD/g in darkness, compared to merely 5.90 ± 1.1 OD/g under white light conditions [21]. The duration of light exposure has a dose–response relationship with fungal growth. Continuous light exposure (such as 24 h) usually inhibits the mycelial extension rate, while 12 h light–dark alternation can promote the formation of conidia in Botrytis cinerea by activating light-responsive genes such as *brlA* [22]. Light signals affect the stress resistance of fungi by regulating the redox system and cell wall metabolism. Aspergillus fumigatus enhances its resistance to acute ultraviolet and oxidative stress under visible light exposure, accompanied by changes in the expression of cell wall-related genes, showing increased sensitivity to red light. This light response mechanism involves the synergistic action of the blue light receptor *LreA* and the red light receptor *FphA*, where *LreA* regulates mycelial pigment synthesis and *FphA* participates in maintaining cell wall integrity [23]. It is noteworthy that light exposure exerts a dual effect on the development of edible fungal fruiting bodies. Appropriate blue light not only facilitates the formation of fruiting bodies in edible fungi such as *Lentinula edodes* but also enhances the content of vitamin D2 and antioxidant substances, thereby improving nutritional value [24]. These studies elucidate that light signals regulate fungal growth, stress response, and reproductive processes through multiple pathways.

Light, as a pivotal environmental factor, regulates fungal growth, development, and metabolic processes through photoreceptor-mediated signal transduction networks. Upon recognition by photoreceptors such as cryptochromes (*CRYs*), blue light signals activate the differential expression of the *BLISTER* (*BLI*) gene family [25,26]. These blue light-responsive genes modulate the transcription of downstream target genes through chromatin remodeling, wherein *BLI* proteins interact with transcription factors to regulate fatty acid synthesis-related genes by altering histone modification states, thereby influencing the composition and fluidity of cellular membrane lipids [27]. This dynamic remodeling of the membrane system enhances fungal tolerance to oxidative stress and temperature fluctuations, maintaining cellular structural stability [28]. In the *BLI*-mediated regulatory network, the expression patterns of the cytochrome P450 (*CYP*) gene family undergo significant alterations. Light signals, through the *BLI* protein, modulate the transcriptional efficiency of key enzyme-encoding genes such as *CYP51*, thereby influencing the ergosterol biosynthesis pathway. Dysregulation of this pathway can lead to changes in cell membrane permeability [29,30]. Light regulation also extends to mitochondrial energy metabolism pathways. The *BLI* gene affects the expression levels of cytochrome c oxidase assembly proteins (*COX*) by regulating genes associated with the mitochondrial respiratory chain [31]. As a core component of the mitochondrial electron transport chain, changes in COX protein abundance directly impact ATP production efficiency, subsequently regulating hyphal growth rate and fruiting body development [32]. Therefore, light through the *BLI* gene, integrates membrane system remodeling, secondary metabolic regulation, and energy metabolism networks, forming a multi-layered regulatory system that endows fungi with the ability to adapt to complex environments.

Although the regulatory effects of light on fungal growth have been extensively investigated, most previous transcriptomic studies on *Poria cocos* and related medicinal fungi have focused on responses to specific light qualities rather than the quantitative variation in light duration, transcriptional changes induced by abiotic stresses or nutrient conditions with limited attention to light signal transduction pathways, and the identification of differential genes without constructing systematic regulatory networks [33]. In contrast, this study sets light exposure duration as the sole variable to fill the gap in quantitative analysis of dynamic gene expression in *Poria cocos* under different photoperiods. By integrating RNA sequencing with weighted gene co-expression network analysis, we aim to not only identify differential genes but also uncover core regulatory modules and potential signaling axes, with transcriptional results validated via qRT-PCR. Additionally, we link molecular changes to key physiological traits to establish a comprehensive connection between genotype and phenotype. This study intends to systematically elucidate the molecular mechanism by which light duration regulates *Poria cocos* growth activity, thereby providing innovative theoretical support for optimizing its cultivation strategies.

## 2. Materials and Methods

### 2.1. Plant Materials

The *Poria cocos* materials used in the experiment were collected from a *Poria cocos* cultivation base in Banshan Village, Meixian Town, Youxi County, Sanming City, Fujian Province (117°48′30″–118°40′ E, 25°50′36″–26°26′30″ N). The area is characterized by sandy soil along the riverbank, with a pH of 5.3. It has a subtropical monsoon humid climate, with an average annual temperature of 19.2 °C, an annual precipitation of approximately 1600 mm, a frost-free period of 312 days, and an average annual sunshine duration of about 1700 h. The *Poria cocos* strain C21 was obtained from the Fungi Research Institute of Sanming City. It was purified and rejuvenated by the Jiangxi Provincial Forestry Research Institute to produce the mother culture and the original culture, and then cultivated into the cultivation strain by Fujian Forest Home Ecological Development Co., Ltd. (Sanming, China). It was inoculated onto pine logs in mid-July 2023. In May 2024, *Poria cocos* samples with light exposure durations of 0 days (DT0, sample 1), 15 days (DT15, sample 2), and 30 days (DT30, sample 3) were selected on-site. The light treatment relied on natural sunlight (under open-field cultivation conditions without artificial shading or supplementary lighting).The relative humidity of 50 ± 5% (maintained via periodic misting). Three independent culture batches were set up as biological replicates. Five samples were collected from the same tissue site (middle layer) in each batch and mixed into one composite sample for subsequent experiments (RNA extraction, qRT-PCR and physiological analysis). Therefore, all analyses (RNA-seq, qRT-PCR and physiological index detection) were based on three composite samples. Samples were taken from the same part of the *Poria cocos* using a sterile surgical knife, rinsed with sterile water to remove soil impurities, placed in plastic bags, numbered, and stored in liquid nitrogen for subsequent laboratory analysis. Transcriptome sequencing was conducted on the three groups of *Poria cocos* samples, and the differentially expressed genes were compared and analyzed. The comparison strategy was: 15 days/0 days (covered with soil): 2 vs. 1; 30 days/0 days (covered with soil): 3 vs. 1.

### 2.2. RNA-Seq Sequencing and Transcriptome Assembly

Under different light exposure conditions, the ethanol precipitation method and CTAB-PBIOZOL were used to enrich mRNA with polyA tails through Oligo(dT) magnetic beads, and total RNA was extracted. Double-stranded cDNA was synthesized, and the 3′ end repair and dA-Tailing were completed. Sequencing adapter ligation was carried out, and libraries with insert fragments of 250–350 bp were obtained. Nine cDNA libraries were constructed by PCR amplification. After quality control of the libraries, the libraries were summarized based on the target sequencing data, and sequencing was performed using the Illumina platform, generating 150 bp paired-end reads. The raw data was filtered using fastp (v0.23.2), mainly removing reads with adapters; when the N content in any sequencing read exceeded 10% of the base count of that read, this paired read was removed; when the number of low-quality (Q ≤ 20) bases in any sequencing read exceeded 50% of the base count of that read, this paired read was also removed. All subsequent analyses were based on clean reads. The Trinity [34] program was used to assemble the transcript sequences of this species, and then Corset was used to remove redundancies to obtain Unigene sequences. TransDecoder (https://github.com/TransDecoder/TransDecoder/wiki, accessed on 19 November 2025) was used to predict CDS from the transcripts assembled by Trinity, and the amino acid sequences corresponding to the transcripts were obtained.

### 2.3. Analysis of Differential Expressed Genes

This study employed a de novo transcriptome approach. The expression levels of transcripts were calculated using RSEM v1. 3. 1, and FPKM values for each transcript were determined based on transcript length [35]. DESeq2 [36,37] was used to conduct differential expression analysis between sample groups, thereby obtaining the differentially expressed gene sets between the two biological conditions. The *p*-values were corrected using the Benjamini & Hochberg method. The corrected *p*-values and |log2foldchange| were used as thresholds for significant differential expression. The screening criteria for differentially expressed genes were |log2Fold Change| ≥ 1 and FDR < 0. 05. The non-redundant transcript sequences were compared with the KEGG, NR, Swiss-Prot, GO, COG/KOG, and Trembl databases using DIAMOND v2. 0. 9 [38], and the amino acid sequences were compared with the Pfam database using HMMER 3. 2 to obtain annotation information for the transcripts in the seven major databases. Enrichment analysis was performed based on hypergeometric tests, with KEGG at the pathway level and GO based on GO terms.

### 2.4. WGCNA Constructs Co-Expression Modules and Regulatory Networks

During the implementation of weighted gene co-expression network analysis (WGCNA), version 1.71 of the tool was used. The specific parameter settings are as follows: the module merging threshold (mergeCutHeight) was set to 0.25, the goodness-of-fit threshold (RsquaredCut) was set to 0.85, the topological overlap matrix type (TOMType) was set to the “signed” mode, and the minimum module size (minModuleSize) was determined to be 50. Additionally, the visualization of the regulatory network was accomplished using Cytoscape v3. 10. 2 software.

### 2.5. Real-Time Fluorescence Quantitative Reverse Transcription Polymerase Chain Reaction (qRT-PCR) Analysis

Several DEGs associated with the growth and metabolism of *Poria cocos* under different light exposure times were selected for qRT-PCR verification. The qRT-PCR reaction was performed using a PCR detection system (Bio-Rad, Hercules, CA, USA) and SYBR Green Master Mix (Takara, Dalian, China). The reaction mixture contained 1 μL cDNA template, 10 μL 2 × SYBR Green Master Mix, and 1 μL of each primer (10 μmol/μL), with water added to a final volume of 20 μL. The amplification program consisted of one cycle at 95 °C for 30 s, followed by 39 cycles of 95 °C for 5 s, TM for 20 s, and 72 °C for 20 s. *His 3-1* was used as the internal reference gene. All qPCR detections were conducted with three biological replicates and four technical replicates, and the quantitative analysis was performed using the 2^−ΔΔCT^ method [39]. The significance mark: The same letter indicates an insignificant difference (*p* > 0.05), while different letters indicate an extremely significant difference (*p* < 0.05).

### 2.6. Detection of Physiological Indicators of Poria cocos

The determination of the lignin content in *Poria cocos* was carried out using the spectrophotometric method. After the lignin reaction, the acetylated lignin produced has a characteristic absorption peak at 280 nm. The lignin content can be quantified by the change in absorbance value. The specific steps can be referred to the experimental method of Xiaozhi Ma et al. [40]. The total acid was determined by the national standard acid-base titration method. According to the principle of acid-base neutralization, the acid in the test solution was titrated with alkali solution, and the endpoint of the titration was determined by the phenolphthalein indicator. The total acid content in the sample was calculated based on the consumption of the alkali solution [41].After the total sugar was hydrolyzed, it was heated with DNS reagent under alkaline conditions and reduced to amino compounds. It turned red-brown in an alkaline solution and had a linear relationship with the absorbance at 540 nm within a certain concentration range. The total sugar content in the sample was determined based on the standard curve [42].

## 3. Results

### 3.1. Identification and Functional Annotation of Poria cocos Transcriptome Unigene Under Different Light Exposure Times

**Figure 1:**
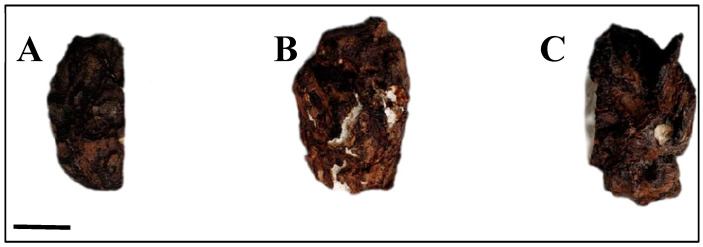
Samples of *Poria cocos* under different light exposure durations. (**A**): 0 day; (**B**): 15 days; (**C**): 30 days. Scale bar = 10 cm.

Sequencing was conducted on the *Poria cocos* samples of DT0, DT15, and DT30. The PCA analysis of the samples showed good repeatability and clear grouping (Figure 1). A total of 509,312,046 raw reads were generated (Table 1). After removing low-quality sequences, 450,909,790 clean reads were obtained. The clean reads were used for transcript assembly. The average sequence length of the transcripts was 2610 bp, with an N50 length of 4723 bp and an N90 length of 1348 bp. The average sequence length of single genes was 3198 bp, with an N50 length of 5028 bp and an N90 length of 1621 bp (Table 2).

**Figure 2:**
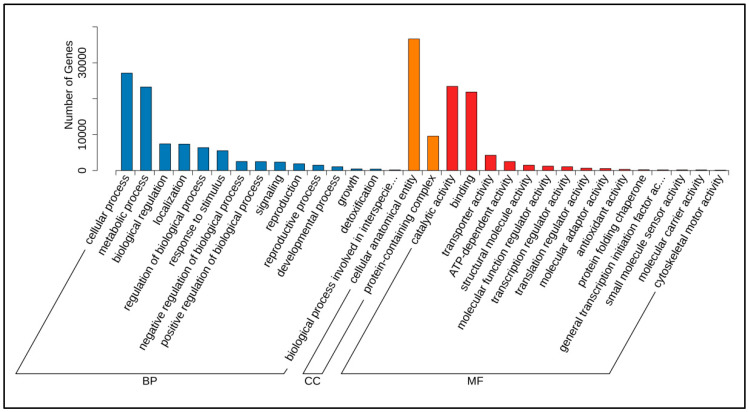
Bar chart of Gene Ontology (GO) classification.

In this study, we conducted a comprehensive functional analysis of total unigenes through annotation across seven databases (Table S1). Among the total unigenes, 58,622 were annotated across all databases, with 54,101 (92.29%) successfully annotated in at least one database. Following Gene Ontology (GO) annotation, the successfully annotated genes were systematically classified into subcategories of the three primary GO domains: Biological Process, Cellular Component, and Molecular Function (Figure 2). Our analysis revealed significant enrichment of genes associated with “cellular processes,” “metabolic processes,” “single-organism processes,” and “biological regulation,” providing valuable insights into the specific developmental mechanisms of *Poria cocos*.

### 3.2. Identification and Characterization of Total Differentially Expressed Genes (DEGs) in Poria cocos Under Different Light Exposure Times

**Figure 3:**
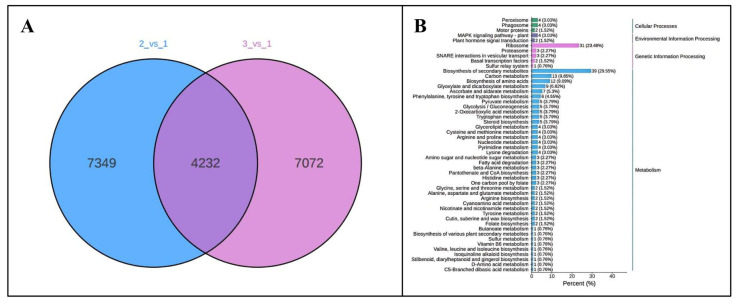
Analysis of differentially expressed genes of *Poria cocos* under different light conditions. (**A**): Venn diagram of differentially expressed genes in different groups; (**B**): KEGG annotation of differentially expressed genes.

To investigate the changes in mRNA expression of *Poria cocos* under different photoperiodic stimuli, the total differentially expressed genes (DEGs) were identified and annotated. In the 2_vs_1 comparison, 11,581 DEGs were identified, comprising 5126 upregulated and 6455 downregulated genes. Similarly, the 3_vs_1 comparison revealed 11,304 DEGs, including 6001 upregulated and 5303 downregulated genes (Table S2). A total of 4332 DEGs were identified in the pairwise comparison(2_vs_1 vs. 3_vs_1) (Figure 3A). Through transcriptomic analysis of *Poria cocos* DEGs, KEGG pathway enrichment analysis (Figure 3B; Table S3) demonstrated significant enrichment of DEGs in secondary metabolite biosynthesis pathways (29.55%). The extensive activation of genes related to phenylpropanoid, terpenoid, and alkaloid biosynthesis revealed the molecular basis of active component synthesis in *Poria cocos*. The high expression of ribosomal-related genes (23.48%) indicated an active state of protein synthesis in *Poria cocos* cells, potentially associated with rapid mycelial growth and efficient synthesis of secondary metabolites. The significant enrichment of carbon metabolism (9.85%) and amino acid biosynthesis pathways (9.09%) reflected the efficient utilization of carbon sources through glycolysis/gluconeogenesis (3.79%) and the glyoxylate cycle (6.82%) in *Poria cocos*.

In the context of energy metabolism-related pathways (Figure 3B), the differential expression of genes involved in pyruvate metabolism (3.79%), glycerolipid metabolism (3.03%), and fatty acid degradation (2.27%) collectively constitute the unique energy metabolism network of *Poria cocos*. Notably, the activation of the ascorbate metabolism pathway (5.3%) may be implicated in the regulation of the antioxidant system in *Poria cocos*. The enrichment of the MAPK signaling pathway (3.03%) and the plant hormone signal transduction pathway (1.52%) suggests that *Poria cocos* may respond to external environmental stimuli through a conserved signal transduction system. Among the genes related to organelle function, the active expression of peroxisomes (3.03%) and the vacuolar transport system (2.27%) may be associated with the storage and transport of its secondary metabolites. Within the genetic information processing system, the synergistic action of ribosome biogenesis (23.48%) and the proteasome (2.27%) collectively maintains the precise regulation of protein homeostasis in *Poria cocos*. The identification and characterization of these differentially expressed genes across multifunctional pathways reveal that the changes in gene expression in *Poria cocos* under different light treatments extensively encompass biological processes such as metabolic regulation, protein synthesis, and signal transduction, providing critical genetic insights into the molecular mechanisms by which light duration influences the growth activity of *Poria cocos*.

### 3.3. Differentially Expressed Genes (DEGs) in Response to Photoperiodic Stimulation in Poria cocos

**Figure 4:**
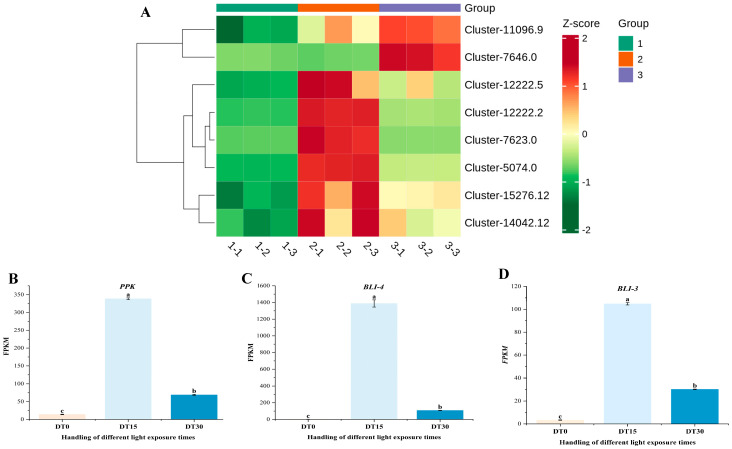
Heatmap of some differentially expressed genes related to the light response of *Poria cocos*. (**A**) Heatmap of the expression of key genes related to light response in different samples; (**B**) The FPKM values of the *PPK* gene in different samples; (**C**) The FPKM values of the *BLI-4* gene in different samples; (**D**) The FPKM values of the *BLI-3* gene in different samples. (Errorline: ±standard error, represents the degree of data dispersion; Significance mark: The same letter indicates an insignificant difference (*p* > 0.05), while different letters indicate a significant difference (*p* < 0.05)).

Among the 4233 differentially expressed genes identified, the dynamic expression profiles of *Poria cocos* light signal-responsive genes under continuous illumination treatment are illustrated (Figure 4A). The *FYPP3* gene maintains basal expression levels under dark conditions, exhibiting an initial significant upregulation followed by a moderate decline upon light induction. Members of the *PPK* gene family demonstrate heterogeneous regulatory patterns, with Cluster-12222.2 showing explosive activation at 15 days of light stimulation, achieving a 23.98-fold increase in FPKM value, thereby emerging as the most prominent light-responsive regulatory factor (Figure 4B). Other homologous genes display either progressive or sustained accumulation-type expression characteristics. The *RPT* gene reaches its expression peak during mid-term light treatment, maintaining a stable high-expression state until the treatment’s conclusion. Notably, the *BLI-4* gene family members exhibit extreme light-responsive properties: Cluster-7623.0 demonstrates a dramatic increase from an initial FPKM value of 2.41 to 1388.35 after 15 days of light treatment, representing a 576.08-fold amplification, making it the gene with the most drastic expression change in the entire dataset (Figure 4C). Its homologous gene *BLI-3*, Cluster-5074.0, also shows a significant 31.27-fold upregulation, collectively constituting the core response elements of the *Poria cocos* light signal transduction system (Figure 4D). The *WC1* gene displays a unique delayed response pattern, showing significant activation at the 30-day treatment endpoint. These temporal expression data systematically elucidate the hierarchical characteristics of the gene regulatory network in *Poria cocos* light signal transduction, particularly highlighting the hypersensitive response properties of *BLI-4* gene family members under intense light stimulation, providing crucial targets for deciphering the molecular mechanisms of *Poria cocos* light response.

### 3.4. Regulation of Differential Genes in the Growth and Metabolic Pathways of Poria cocos by Light Duration

**Figure 5:**
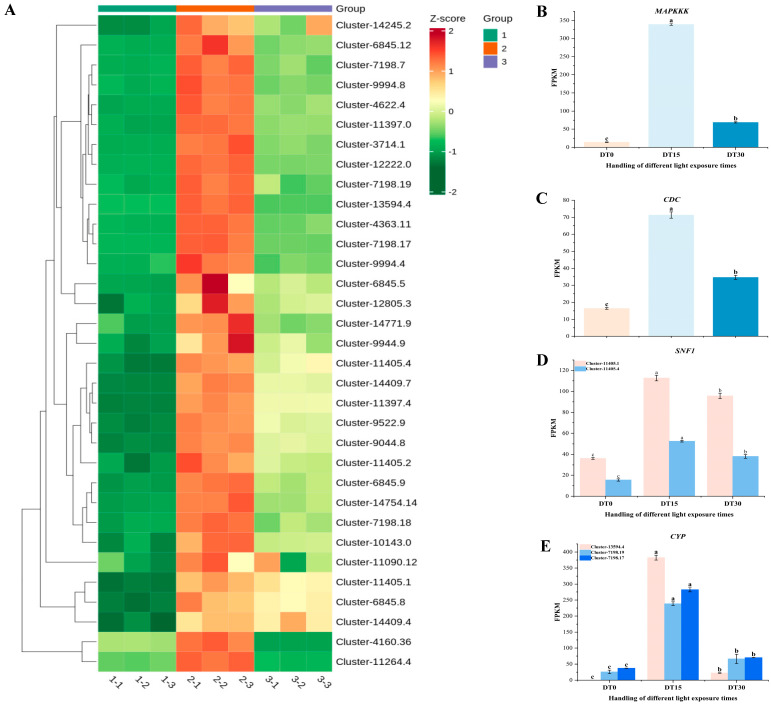
Heatmap of some differentially expressed genes related to the metabolic pathways of *Poria cocos. *(**A**) Heatmap of the expression of key genes related to metabolic pathways in different samples; (**B**) FPKM values of the *MAPKKK* gene in different samples; (**C**) FPKM values of the *CDC* gene in different samples; (**D**) FPKM values of the *SNF1* gene in different samples; (**E**) FPKM values of the *CYP* gene in different samples. (Errorline: ±standard error, represents the degree of data dispersion; Significance mark: The same letter indicates an insignificant difference (*p* > 0.05), while different letters indicate a significant difference (*p* < 0.05)).

Based on the information annotated in KEGG, we identified a series of genes related to the growth and metabolic pathways of *Poria cocos* from the DEGs (Figure 5A). Among these DEGs, *MAPK*, *MAPKKK*, *CYP*, *SNF1*, *CDC*, *HOG1*, *COX*, and *GCN2*, which are related to fungal growth and metabolism, showed different expression patterns under different light stimulation times. They were highly expressed at DT 15 but lowly expressed at the DT 0 and DT 30. The core component of the MAPK signaling pathway, *MAPKKK* (Cluster-12222.0), was activated explosively after 15 days of light exposure, with its expression level increasing 28.11 times compared to the initial level, becoming the most significant regulatory factor in this pathway (Figure 5B). Its downstream effector molecule *MAPK* (Cluster-14409.7) and the *HOG1* homolog (Cluster-14409.7) worked together to form the core transmission module for environmental signal perception. The key gene for cell cycle regulation, *CDC* (Cluster-14754.14), reached a 4.34-fold expression peak at the mid-term of light exposure, suggesting that light may affect the growth rate of mycelia by regulating the cell division cycle (Figure 5C).

In the energy metabolism network, the *SNF1* homolog (Cluster-11405.1) was stably upregulated by 3.11 times under continuous light exposure (Figure 5D), and its homolog Cluster-11405.4 showed a 3.40-fold increase in expression, jointly regulating carbon source metabolism and energy balance. The *COX* gene (Cluster-6845.8), as a core component of the mitochondrial electron transport chain, was upregulated by 5.53 times at the mid-term of light treatment, significantly higher than other members of the same family. Members of the cytochrome *P450* (*CYP*) superfamily exhibited extreme light response characteristics (Figure 5E). Among them, Cluster-13594.4 showed a 5464-fold increase in expression at 15 days, becoming the most variable metabolic-related gene; Cluster-7198.19 and Cluster-7198.17 were significantly upregulated by 9.25 and 7.55 times, respectively, jointly constituting the core catalytic system of the terpene and alkaloid synthesis pathways. These temporal expression characteristics systematically analyzed the hierarchical structure of the light-regulated metabolic network of *Poria cocos*, especially the core role of *CYP* superfamily members in the synthesis of secondary metabolites, providing a molecular basis for clarifying the light-adaptive metabolic mechanism of *Poria cocos*.

### 3.5. DEG Response of Cell Damage in Poria cocos Under Different Light Time Stimulation

**Figure 6:**
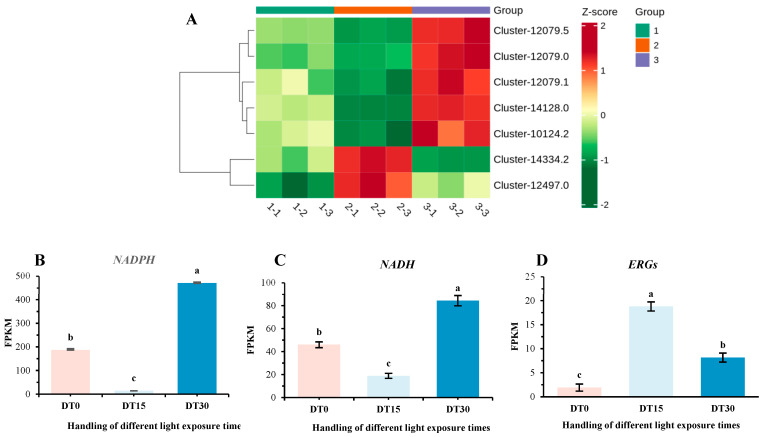
Heatmap of some differentially expressed genes related to cell damage and cell membrane homeostasis in *Poria cocos.* (**A**) Heatmap of the expression of key genes related to cell damage and cell membrane homeostasis in different samples; (**B**) FPKM values of the *NADPH* gene in different samples; (**C**) FPKM values of the *NADH* gene in different samples; (**D**) FPKM values of the *ERGs* gene in different samples. (Error line: ±standard error, represents the degree of data dispersion; Significance mark: The same letter indicates an insignificant difference (*p* > 0.05), while different letters indicate a significant difference (*p* < 0.05)).

Common reactive oxygen species (*ROS*) include singlet oxygen, superoxide, hydroxyl radicals, and hydrogen peroxide [43]. These *ROS* readily react with lipids, DNA, proteins, and other macromolecules, leading to cell death and aging, thereby significantly impacting the growth and vitality of organisms (Figure 6A). Our research has identified that the differentially expressed genes (DEGs) associated with the growth and metabolism of *Poria cocos* exhibit high expression levels at the DT 15 stage and low expression levels at the DT 0 and DT 30 stages. We hypothesize that prolonged light exposure may cause certain damage to *Poria cocos*, subsequently affecting its growth and metabolic capabilities, and ultimately exerting adverse effects on its overall growth status. To investigate this, we examined the expression of genes involved in cellular damage and membrane homeostasis in *Poria cocos* under varying light exposure durations. We identified several DEGs, including *NADPH*-dependent oxidoreductases (Figure 6B), the *NADH* gene family (Figure 6C) and the *ERG* gene family (Figure 6D). These DEGs are associated with *ROS* production and membrane homeostasis. Genes related to *ROS* production showed high expression levels at the DT 0 and DT 30 stages, with a significant increase at the DT 30 stage, and low expression at the DT 15 stage. Conversely, genes related to membrane homeostasis exhibited high expression at the DT 15 stage and low expression at the DT 0 and DT 30 stages. This indicates that under different light exposure durations, *Poria cocos* generates a higher amount of *ROS* at the DT 0 and DT 30 stages, leading to severe cellular damage and affecting its growth activity. However, at the DT 15 stage, the synthesis of ergosterol increases, enhancing membrane integrity and enabling better resistance to external factors such as osmotic pressure changes and chemical erosion. Additionally, the increased synthesis of ergosterol maintains membrane fluidity, ensuring normal material exchange and signal transduction across the membrane, which facilitates nutrient absorption and metabolic waste excretion, thereby improving the growth activity of *Poria cocos*.

### 3.6. Analysis of Coexpression Network of DEG WGCNA Associated with Growth and Metabolism of Poria cocos

**Figure 7:**
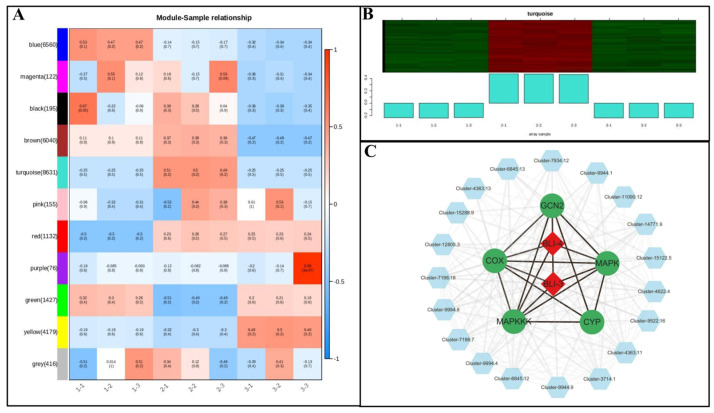
WGCNA of differentially expressed genes in *Poria cocos*. (**A**): Heatmap of the correlation between samples and modules; (**B**): Diagram of the gene expression patterns of modules; (**C**): Regulatory network diagram of key genes in *Poria cocos* under light stimulation.

To elucidate the synergistic regulatory relationships of differentially expressed genes (DEGs) in *Poria cocos* under varying photoperiods, this study employed Weighted Gene Co-expression Network Analysis (WGCNA) to construct a gene co-expression network by selecting DEGs with correlation coefficients greater than 0. 5 across various pathways, thereby focusing on core regulatory modules and key hub genes. The results (Figure 7A) demonstrated that all DEGs were clustered into multiple co-expression modules, among which the turquoise module exhibited the most significant biological relevance (Figure 7B). This module comprised 237 core genes, showing the highest positive correlation with the 15-day photoperiod treatment (DT15) (correlation coefficient r = 0.87, *p* < 0.01), indicating its pivotal role as the core functional module in *Poria cocos*’ response to the 15-day photoperiod.

Within the key node genes contained in the module, *CYP* family members such as Cluster-13594.4 and Cluster-7198.17 exhibited significantly high expression levels during the DT15 phase. Notably, the expression level of Cluster-13594.4 was upregulated by 5464-fold compared to the 0-day light exposure (DT0), representing the most dramatic expression change among the metabolism-related genes in the module, primarily involved in the biosynthesis of secondary metabolites such as ergosterol. The blue light-responsive gene *BLI-4* (Cluster-5074.0) showed a 31. 30-fold upregulation during the DT15 phase, serving as a core element in light signal perception and playing a pivotal role in connecting external light stimuli with internal metabolic regulation. Meanwhile, *MAPKKK* (Cluster-12222.0), as an upstream regulatory factor in the *MAPK* signaling pathway, demonstrated a 28.11-fold increase in expression during DT15 compared to DT0, responsible for transmitting light signals through cascade reactions to downstream effector molecules. Functional enrichment analysis of genes within the turquoise module further revealed significant enrichment in secondary metabolite synthesis pathways and redox processes. These findings are highly consistent with the KEGG enrichment analysis results, which indicated that differentially expressed genes were primarily involved in secondary metabolite synthesis and carbon metabolism pathways, thereby fully validating the module’s central role in *Poria cocos* substance synthesis and environmental adaptation.

*BLI-4* exhibits a robust interaction with the *CYP* gene family (Figure 7C), suggesting that under 15-day photoperiod stimulation, blue light signaling may directly regulate the transcriptional activity of *CYP* family genes through the activation of *BLI-4* expression. As key enzyme-encoding genes in the ergosterol biosynthesis pathway, the activation of *CYP* family genes promotes ergosterol synthesis, thereby enhancing cell membrane stability. This finding aligns with previous observations of elevated expression of ergosterol synthesis genes (*ERG* family) and increased cell membrane stability during the DT15 stage, thus completing the regulatory cascade. Furthermore, the high correlation between *MAPKKK* and *CYP* family members indicates that the *MAPK* signaling pathway may participate in the cascade regulation of photoresponsive genes, amplifying signals through phosphorylation and other modifications to enhance the regulatory efficiency of *CYP* family genes. We propose the hypothesis of the “*BLI-4-MAPKKK-CYP*” regulatory axis, which is grounded in co-expression correlations and would benefit from further functional validation to confirm potential causal relationships.

### 3.7. Some DEG Related to Growth and Metabolism of Poria cocos Was Verified by qRT-PCR

**Figure 8:**
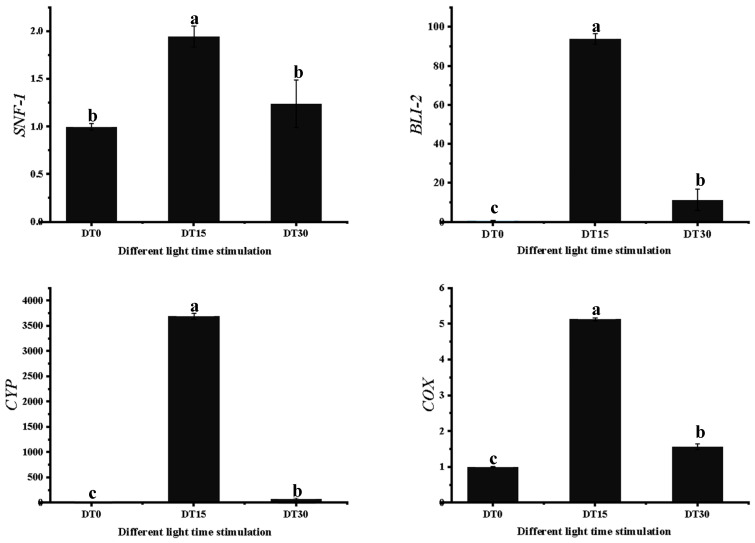
Validation of four candidate genes of *Poria cocos* by quantitative real-time reverse transcription polymerase chain reaction (qRT-PCR). (Error line:±standard error, represents the degree of data dispersion; Significance mark: The same letter indicates an insignificant difference (*p* > 0.05), while different letters indicate a significant difference (*p* < 0.05)).

In independent biological experiments utilizing qRT-PCR, the transcriptional regulation revealed by RNA-seq was validated. Gene-specific primers were designed for four selected genes across three developmental stages, with his 3-1 (Histone 3) serving as the internal reference gene. The qRT-PCR validation results exhibited a high degree of consistency with the RNA-seq data trends. Four differentially expressed genes (*SNF*, *CYP*, *BLI*, *COX*) were selected for primer design (Table S4) and subsequent expression analysis (Figure 8). The results demonstrated that the expression patterns of all examined genes showed significant correlation with transcriptomic data. Specifically, the transient high expression of the *CYP* gene in the 15-day treatment group precisely matched the dynamic changes in CYP family members observed in RNA-seq. The expression surge of the *BLI* gene at 15 days aligned with the response pattern of *BLI-4*, while the expression trends of *SNF* and *COX* genes showed high concordance with the transcriptional characteristics of *SNF* homologs and *COX* genes, respectively. These findings indicate excellent reproducibility between transcript abundance detected by RNA-seq and expression profiles revealed by qRT-PCR, thereby validating the reliability of transcriptomic analysis results and providing experimental evidence for the molecular mechanisms underlying light duration regulation of *Poria cocos* growth metabolism.

### 3.8. The Changes in Physiological Indicators of Poria cocos Under Different Light Exposure Times

**Figure 9:**
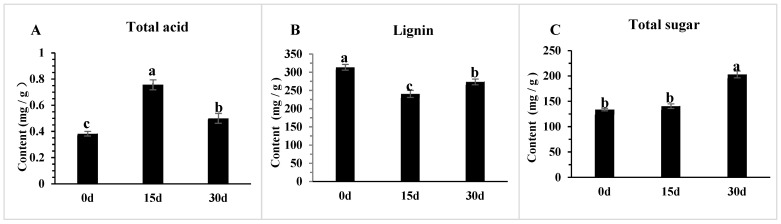
The physiological index changes for Poria *cocos.* (**A**) Total acid content in different samples; (**B**) Lignin content in different samples; (**C**) Total sugar content in different samples. (Error line: ±standard error, represents the degree of data dispersion; Significance mark: The same letter indicates an insignificant difference (*p* > 0.05), while different letters indicate a significant difference (*p* < 0.05)).

Under different light treatment durations (0 days, 15 days, 30 days), the total acid content, lignin content, and total sugar content of *Poria cocos* showed significant differences. Moreover, the dynamic fluctuation characteristics of each index at key time points were closely related to the physiological and metabolic responses regulated by light. The total acid content reached its peak at 15 days of light treatment, with a value of 0.76 mg/g, which was approximately 1.97 times higher than the initial 0.38 mg/g at 0 days; subsequently, it dropped to 0.50 mg/g at 30 days, although it decreased by 33.98% compared to 15 days, it was still 30.55% higher than the initial level at 0 days (Figure 9A).

The lignin content showed different trends of change (Figure 9B). At 15 days, the lignin content dropped to 240.99 mg/g, which was 23.24% lower than the 313.96 mg/g at 0 days; at 30 days, the lignin content rose to 274.04 mg/g, increasing by 13.72% compared to 15 days, but still 12.72% lower than the initial level at 0 days.

The total sugar content showed a continuous upward trend with the extension of light exposure time (Figure 9C). At 30 days, the total sugar content reached 203.38 mg/g, which was approximately 1.52 times higher than that at 0 days (134.11 mg/g), and increased by 44.50% compared to 15 days (140.76 mg/g). This indicates that extending the light exposure time significantly promotes the accumulation of total sugar in *Poria cocos*, and the accumulation effect at 30 days is much higher than that at 15 days.

In summary, the regulatory effects of different light exposure times on the physiological indicators of *Poria cocos* vary significantly. Short-term light exposure (15 days) mainly promotes the accumulation of total acid, while long-term light exposure (30 days) is more conducive to the significant accumulation of total sugar. This provides data support for optimizing the light management strategy in *Poria cocos* cultivation and directing the regulation of its effective component accumulation.

## 4. Discussion

Light serves as a pivotal signaling element in the growth and development of fungi [44]. In this study, two blue light rapidly induced genes, *BLI-4* and *BLI-3*, were identified in *Poria cocos*. When exposed to varying durations of light stimulation, these genes exhibited remarkably distinct and significant response characteristics, with their transcriptional levels demonstrating a sensitive and adaptive regulation in accordance with the dynamic changes in light exposure. Specifically, during the DT 15 phase, the transcriptional levels of both genes displayed a pronounced upward trend; however, at DT 0 and DT 30 phases, their transcriptional levels declined. Previous research has established that *BLI-4* and *BLI-3*, as blue light rapidly induced genes, exhibit a ninety-fold increase in transcriptional rate under blue light irradiation compared to dark control levels. Additionally, the proteins encoded by *BLI-4* and *BLI-3* are rapidly transported to the mitochondria and exhibit high homology with the short-chain alcohol dehydrogenase family [45]. Based on existing studies, we hypothesize that the proteins encoded by *BLI-4* and *BLI-3* likely play a crucial role in the mitochondria of *Poria cocos* cells, with this function being closely linked to physiological processes such as energy metabolism under varying light exposure durations. Specifically, during the DT 15 phase, *Poria cocos* cells exhibit a robust response to light stimulation signals, and the expression products of *BLI-4* and *BLI-3* may, through a series of complex molecular mechanisms, effectively promote the growth and development of *Poria cocos* cells, enhance cellular energy metabolism efficiency, and positively influence other physiological processes involving mitochondria. Conversely, during the DT 0 and DT 30 phases, the situation differs, with *BLI-4* and *BLI-3* genes and their encoded proteins potentially exerting inhibitory effects on the aforementioned physiological processes. Overall, *BLI-4* and *BLI-3* genes play a critical role in connecting light signals with cellular physiological functions in *Poria cocos* cells. The transient high expression of *BLI-4* during the DT 15 phase may enhance cell membrane stability through the short-term activation of ergosterol synthesis, while prolonged light exposure (DT 30) may trigger negative feedback inhibition, leading to downregulation of its expression. This pattern resembles the response mechanism of *BLI* genes in *Neurospora crassa* [], and the specific regulatory mechanisms warrant further in-depth investigation.

Current research predominantly focuses on the impact of light quality on the metabolism of medicinal fungi. However, there is a lack of reported studies on the regulation of the gene network in *Poria cocos* by light duration. Based on our own research results, this study further compared and verified the research results of others regarding the key genes related to the physiological processes of fungal growth, development, and metabolism. It was found that the two sets of results were highly consistent. Eventually, multiple such key genes were identified, including *cytochrome P450 (CYP)* [46], *SNF1* [47], *CDC42* [48], *HOG1* [49,50], *COX* [51], *MAPK*, *MAPKKK* [52,53], and *GCN2* [54]. These key genes exhibit unique expression patterns in the transcriptome of *Poria cocos*, with studies showing high expression levels during the DT 15 phase, while transitioning to low expression levels during the DT 0 and DT 30 phases. This differential expression pattern suggests that they play a distinct and crucial regulatory role in the growth, development, and metabolism of *Poria cocos* under varying light durations. During the DT 15 phase, these genes actively participate in and promote the physiological processes of growth, development, and metabolism in *Poria cocos*, demonstrating significant positive regulatory efficacy and effectively driving the efficient operation of growth, development, and various physiological functions in this phase. Conversely, during the DT 0 and DT 30 phases, their regulatory roles shift, exerting a decelerating effect on the growth, development, and metabolic processes of *Poria cocos*. We have discovered the intrinsic mechanism behind the specific expression patterns of genes closely related to fungal growth, development, and metabolism; namely, these genes exhibit high expression levels during the DT 15 phase and low expression levels during the DT 0 and DT 30 phases. To elucidate this specific expression pattern, we screened and identified differentially expressed genes (DEGs), pinpointing genes closely associated with reactive oxygen species (*ROS*) generation and cell membrane homeostasis, such as *NADPH*-dependent oxidoreductase, the *NADH* gene family, and the ERG gene family. The expression changes of NADPH-dependent oxidoreductase under different light durations have a critical impact on the production of *ROS* in *Poria cocos*. During the DT 0 and DT 30 phases, its high expression leads to a substantial generation of *ROS*. It is hypothesized that excessive *ROS* can attack biological macromolecules such as lipids, proteins, and DNA within cells, causing damage to cell structure and function, thereby inhibiting the expression of genes related to growth and metabolism in *Poria cocos* and affecting its growth and development. In contrast, at DT15, enhanced ergosterol synthesis may improve membrane stability, which is a potential mechanism for resisting oxidative stress—this assumption requires direct biochemical measurements in the future to verify.

Based on the data showing the effects of light treatment on the physiological indicators of *Poria cocos*, the dynamic changes in total acid, lignin, and total sugar content collectively reveal the phased characteristics of light regulating the growth of *Poria cocos*, which are closely linked to the molecular changes in *ROS* generation and ergosterol synthesis. As key indicators reflecting the metabolic state of *Poria cocos*, the total acid content reached 0.76 mg/g after 15 days of light treatment, a significant increase of nearly 2 times compared to the initial 0.38 mg/g at 0 days. This elevation is attributed to the transient high expression of *BLI-4* and *BLI-3* genes at the DT15 stage, which not only activates ergosterol synthesis (via upregulation of ERG family genes) to enhance cell membrane stability but also suppresses excessive ROS production by downregulating NADPH-dependent oxidoreductases. Total acid directly participates in core metabolic processes such as intracellular substance transformation and enzyme activity regulation necessary for growth. The doubling of its content indicates that the metabolic activities of *Poria cocos* cells have been comprehensively enhanced under the favorable molecular environment of intact membrane integrity and reduced oxidative damage [55]; however, after 30 days of light treatment, the total acid content dropped to 0.50 mg/g. Although it was still 31.58% higher than the initial level at 0 days, it decreased by 34.21% compared to the peak value at 15 days. This decline is associated with the downregulation of *BLI-4* and *ERG* family genes under prolonged light exposure, leading to decreased ergosterol synthesis, compromised membrane stability, and accumulated *ROS* that inhibits the activity of growth-related metabolic enzymes, reflecting a significant slowdown in metabolic activity and growth rhythm. Looking at the lignin content related to the cell wall structure, the lignin content under light treatment for 15 days dropped to 241.00 mg/g, which was 23.25% lower than 314.00 mg/g at 0 days. Under the optimal molecular state of DT15, *Poria cocos* prioritizes nutrient allocation to growth-related processes such as cell mass proliferation and polysaccharide synthesis [56], rather than strengthening the cell wall [57]; thus, reducing the proportion of lignin allocated while under light treatment for 30 days, the lignin content rebounded to 274.00 mg/g, an increase of 13.69% compared to 15 days. This rebound is due to the impaired molecular environment that slows growth rate, prompting *Poria cocos* to redirect nutrients from growth to cell wall maintenance for stress resistance. The total sugar content, which serves as the core energy source for the growth of *poria cocos*, shows a change that further refines this growth regulation process from the perspective of energy metabolism, with its changes closely tied to molecular-mediated growth dynamics: during the 15-day light treatment, the total sugar content was 140.80 mg/g, only slightly increasing by 5.00% compared to 134.11 mg/g at day 0. This is because during this stage, *Poria cocos* is in a period of vigorous growth, and the synthesis of total sugar is roughly balanced with the energy consumed by growth. The high energy consumption state precisely corresponds to the rapid growth demand stimulated by light exposure [58]. However, during the 30-day light treatment, the total sugar content significantly increased to 203.38 mg/g, which was 44.45% higher than that after 15 days and 51.65% higher than that at 0 days. At this point, the significant accumulation of total sugar was not due to a sudden increase in synthesis, but rather because the growth rate decreased, resulting in a significant reduction in energy consumption, and the excess sugar shifted from “growth consumption” to “storage and reserve” [59]. The coordinated changes in various physiological indicators of *Poria cocos* indicate that the effect of light on the growth of *Poria cocos* shows a phased process of “stimulating the growth of *Poria cocos* for 15 days, and then inhibiting it for 30 days”.

Comprehensively, the coordinated expression changes in genes associated with reactive oxygen species generation and cell membrane homeostasis collectively constitute the intrinsic regulatory mechanism underlying the specific expression patterns of growth and metabolism-related genes in *Poria cocos* under varying photoperiods. However, our current understanding of the precise regulatory networks among these genes and their interactions with other potential regulatory factors remains insufficient. Future research could further employ gene editing technologies, such as the CRISPR/Cas9 system, to conduct knockout or overexpression experiments on these key genes, directly validating their functions in *Poria cocos* growth metabolism and photoperiod response processes. Simultaneously, by integrating proteomics and metabolomics technologies, a comprehensive analysis of changes in proteins and metabolites of *Poria cocos* under different light conditions could be conducted, providing multi-level insights into the molecular mechanisms by which light influences the growth activity of *Poria cocos*. This will offer a more robust theoretical foundation for the precise cultivation of *Poria cocos* and contribute to the sustainable development of the *Poria cocos* industry.

## Figures and Tables

**Table 1:** The analysis of data output quality. Sample: sample name; Raw Reads: Number of reads in the original data; Clean Reads: Number of high quality reads filtered from original data; Clean Base (G): total number of bases of high quality reads; Q20 (%): percentage of base with phred value > 20; Q30 (%): percentage of base with phred value > 30; GC Content (%): GC ratio of total bases.

Sample	Raw Reads	Clean Reads	Clean Base (G)	Error Rate (%)	Q20 (%)	Q30 (%)	GC Content (%)
DT0 1-1	53,215,642	47,486,284	7.12	0.01	97.86	94.03	57.83
DT0 1-2	54,831,048	49,060,902	7.36	0.01	98.09	94.6	57.79
DT0 1-3	56,642,570	50,319,860	7.55	0.01	98.04	94.47	57.83
DT15 2-1	65,260,872	56,542,448	8.48	0.01	98.06	94.48	57.51
DT15 2-2	55,131,772	48,6182,82	7.29	0.01	97.97	94.27	57.56
DT15 2-3	54,250,752	47,533,692	7.13	0.01	98.21	94.86	57.52
DT30 3-1	68,420,452	61,300,220	9.2	0.01	98.03	94.51	59.2
DT30 3-2	44,341,606	39,574,124	5.94	0.01	97.9	94.18	59.01
DT30 3-3	57,217,332	50,473,978	7.57	0.01	97.95	94.28	59.03

**Table 2:** Assembly result statistics.

Typle	Number	Mean Length	N 50	N 90
Transcript	77,600	2610	4723	1348
Unigene	58,622	3198	5028	1612

## Data Availability

The original contributions presented in this study are included in the article/Supplementary Material. Further inquiries can be directed to the corresponding authors.

## Supplementary Materials

The following supporting information can be downloaded at: https://www.mdpi.com/article/10.3390/genes16121404/s1, Table S1: Annotation Statistics Table; Table S2: Statistical Table of Differential Genes; Table S3: compareKEGG_Enrichment; Table S4: Primers for qPCR.
